# Inhibition of Autophagy Signaling via 3-methyladenine Rescued Nicotine-Mediated Cardiac Pathological Effects and Heart Dysfunctions

**DOI:** 10.7150/ijbs.41275

**Published:** 2020-02-21

**Authors:** Peng Zhang, Yong Li, Yingjie Fu, Lei Huang, Bailin Liu, Lubo Zhang, Xuesi M Shao, Daliao Xiao

**Affiliations:** 1 Lawrence D. Longo, MD Center for Perinatal Biology, Department of Basic Sciences, Loma Linda University School of Medicine, Loma Linda, California, USA; 2 Department of Cardiology, The First Affiliated Hospital of Chongqing Medical University, Chongqing, China; 3 Department of Neurobiology, David Geffen School of Medicine at UCLA, University of California at Los Angeles, Los Angeles, California, USA

**Keywords:** nicotine, cardiac ischemia/reperfusion injury, autophagy pathway

## Abstract

**Rationale:** Cigarette smoking is a well-established risk factor for myocardial infarction and sudden cardiac death. The deleterious effects are mainly due to nicotine, but the mechanisms involved and theranostics remain unclear. Thus, we tested the hypothesis that nicotine exposure increases the heart sensitivity to ischemia/reperfusion injury and dysfunction, which can be rescued by autophagy inhibitor.

**Methods:** Nicotine or saline was administered to adult rats via subcutaneous osmotic minipumps in the absence or presence of an autophagy inhibitor, 3-methyladenine (3-MA). After 30 days of nicotine treatment, the rats underwent the cardiac ischemia/reperfusion (I/R) procedure and echocardiography analysis, and the heart tissues were isolated for molecular biological studies.

**Results:** Nicotine exposure increased I/R-induced cardiac injury and cardiac dysfunction as compared to the control. The levels of autophagy-related proteins including LC3 II, P62, Beclin1, and Atg5 were upregulated in the reperfused hearts isolated from nicotine-treated group. In addition, nicotine enhanced cardiac and plasma ROS production, and increased the phosphorylation of GSK3β (ser9) in the left ventricle tissues. Treatment with 3-MA abolished nicotine-mediated increase in the levels of autophagy-related proteins and phosphorylation of GSK3β, but had no effect on ROS production. Of importance, 3-MA ameliorated the augmented I/R-induced cardiac injury and dysfunction in the nicotine-treated group as compared to the control.

**Conclusion:** Our results demonstrate that nicotine exposure enhances autophagy signaling pathway, resulting in development of ischemic-sensitive phenotype of heart. It suggests a potentially novel therapeutic strategy of autophagy inhibition for the treatment of ischemic heart disease.

## Introduction

Nicotine exposure, either from tobacco cigarette smoking or nicotine use (nicotine gum or patch) has become one of the most pressing public concerns in modern life. Furthermore, electronic cigarette (e-cigarette), an electronic nicotine delivery system has been introduced in the global market during the past ten years. Thus, nicotine addiction and nicotine abuse are becoming a potential public health concern in the 21^st^ century. Epidemiologic studies have shown that cigarette smoking is the most prominent risk factor for coronary heart disease and cardiovascular mortality in the general population 1-4. Although cigarette smoke contains thousands of chemicals, nicotine and its metabolites are recognized as being the major psychoactive compounds and are likely to contribute to the development of cardiovascular disorders. Indeed, clinical and animal studies have suggested that cigarette smoking-induced myocardial ischemia is mediated by nicotine 5-7. However, underlying mechanisms of cardiac dysfunction induced by nicotine exposure are largely unknown and the specific pathophysiologic pathways leading to the development of heart ischemic-sensitive phenotype remain to be identified.

Autophagy is a cellular process associated with unnecessary protein damage and organelle degradation. Autophagy functions as a protein quality controller and cellular homeostasis keeper 8. In the heart, autophagy occurs constitutively at a basal level 9, but is enhanced during pathological conditions, including cardiac hypertrophy, cardiomyopathy, ischemic heart disease and heart failure 10. However, the role of autophagy in ischemic heart injury is controversial and complex 11. Whether autophagy is protective or detrimental is context-dependent. In general, a modest increase in autophagy under ischemic stress appears to be protective, whereas massive activation may be detrimental and induce type 2 cell death (autophagy-induced cell death), which contributes to ischemic cardiac injury 12. Previous studies have shown that cigarette smoke and nicotine exposure cause different tissue and cell damage via activation of autophagy pathway 13-16. This led us to contemplate if chronic nicotine exposure alters the homeostatic autophagy process and exert adverse effects in cardiomyocytes.

Therefore, in the present study we first evaluated whether chronic nicotine exposure increased the heart susceptibility to ischemia/reperfusion (I/R) injury and decreased heart functional recovery after I/R injury. Next, we examined whether chronic nicotine exposure enhanced autophagy-related biomarkers in cardiomyocytes. Finally, we assessed the effect of therapeutic inhibition of autophagy activation on nicotine-mediated cardiac ischemic injury and dysfunction to test the hypothesis that nicotine-mediated autophagy plays a causal role in the development of heart ischemic-sensitive phenotype in an adult rat model. Our findings suggest that autophagy may be a potential therapeutic molecular target for the treatment of cardiovascular ischemic disease.

## Methods

### Experimental animals

The procedures and protocols were approved by the Institutional Animal Care and Use Committee of Loma Linda University. All animal studies followed the guidelines in the National Institutes of Health Guide for the Care and Use of Laboratory Animals. Sprague-Dawley male rats (8-months-old) were purchased from Charles River Laboratories (Portage, MI) and housed individually in Plexiglas acrylic cages, located in air-conditioned rooms (room temperature 22℃, relative humidity 60%; lights on from 8:00 a.m. to 8:00 p.m.). Pellet food and tap water were available *ad libitum*. Rats were anesthetized with isoflurane (5% for induction, 2% for maintenance) and adequate anesthesia was determined by the loss of pedal withdrawal reflex and other reactions from the animals in response to pinching the toe or tail. Saline or nicotine (at 4 µg/kg/min) were individually administered to rats through osmotic minipumps (type 2ML4, Alzet, Durect Corp., Cupertino, CA) and inserted under the skin on the back of rats for 30 days, as previously described in detail 17-19. The dose of nicotine resulted in blood levels closely resembling those occurring in moderate human smokers 20. A total of 45 rats were used in this project and were randomly divided into four groups: 1) saline control (n=13); 2) nicotine (n=10); 3) saline + 3-MA (n=11); and 4) nicotine + 3-MA (n=11). One week before I/R surgery, 3-methyladenine (3-MA) (15 mg/kg/day; Sigma Aldrich, St. Louis, MO) or saline was respectively administered to rats via intraperitoneal (i.p.) injection for seven days. Previous studies have shown that treatment with this dosage of 3-MA significantly inhibits autophagy signaling 21, 22. To separate the confound effect of sex, in this study we only selected male rats in the proposed experiments. In our future studies, we will investigate the effect of nicotine on female rats.

### Echocardiography

To measure LV function and heart chamber dimensions in the four groups of animals, echocardiography was performed with LOGIQ e Ultrasound (GE Medical System) as previously described 23. Briefly, the rats were anesthetized with 2% isoflurane, the chest was shaved, and a layer of acoustic-coupling gel was applied to the thorax. Then the rats were placed in the left lateral decubitus position. M-mode recording of the LV was obtained at the level of the mitral valve in the parasternal view using two-dimensional (2D) echocardiographic guidance in both short and long axis views. Cardiac function and heart dimensions were evaluated by 2D echocardiography on the anesthetized (2% isoflurane) rat. M-mode tracing was used to measure functional parameters such as LV end-diastolic dimension (LVEDD), LV end-systolic dimension (LVESD), LV end-diastolic volume (LVEDV) and LV end-systolic volume (LVESV) were calculated using the above primary measurements and accompanying software. The percentage of LV ejection fraction (EF) was calculated as (LVEDV-LVESV)/LVEDV x 100% and the percentage of LV fractional shortening (FS) was calculated as (LVEDD-LVESD)/LVEDD x 100%. Echocardiography data were recorded and analyzed blindly to the different treatments.

### Myocardial I/R injury model

After 30 days of nicotine treatment, the rats were subjected to ischemia/reperfusion (I/R) procedure *in vivo* as described previously 24, 25. Briefly, rats were anaesthetized with 2% isoflurane and placed on the RoVent Jr. Small Animal Ventilator (Kent Scientific). Ischemia was induced by an occlusion on left anterior descending (LAD) coronary artery for 45 mins. Reperfusion was initiated after 45 mins of ischemia. Myocardial reperfusion was confirmed by changes in the appearance of the heart from pale to bright red. After 24 hours of reperfusion, some of the rats from each group were anesthetized. Their hearts were rapidly removed and serially sectioned along the short axis in 2-mm-thick sections. To measure the infarct size, the slices were then incubated in 2% 2,3,5-triphenyltetrazolium chloride (TTC) solution for ten minutes at 37°C and immersed in formalin for 30 minutes. Viable tissue stained red, while nonviable tissue remained white. The infarct size and the area of LV in each slice were analyzed by computerized planimetry (NIH image J software), corrected for the tissue weight, summed for each heart, and expressed as a percentage of the total left ventricle weight.

### Masson's trichrome staining

Masson's trichrome staining is widely used to study cardiac pathologies including cardiac infarction and fibrosis. In the present study, the heart was rapidly excised, rinsed to remove blood. Then, the heart tissues were cryopreserved by using optimal cutting temperature (OCT) tissue medium and sectioned transversely from the basal part to the apex of left ventricle using a cryostat with 10 µm thickness. Masson's Trichrome staining (Abcam) was performed according to the manufacture's instruction to quantify infarct scar size (blue staining). The infarct scar size was analyzed by computerized planimetry and expressed as the ratio of scar area to total LV area.

### Measurement of superoxide production in heart tissues

The oxidative fluorescent dye hydroethidium (HE) was used to evaluate superoxide production *in situ*, as described previously 19, 26. Briefly, unfixed frozen heart segments were cut into 10-µm thick sections using Leica CM 3050S cryostat at -20℃. The middle slice sections of LV were used and each tissue slides was incubated with HE (5 µM) at 37 ℃ for 30 minutes. Fluorescence in LV sections was obtained with an EVOS FL color imaging system (Life Technologies Corp. Carlsbad, CA). The quantitative analysis of HE fluorescence intensity was analyzed using the NIH Image J software.

### Measurement of reactive oxygen species (ROS) production in plasma

Plasma was collected from the rat after seven days of reperfusion. The total ROS levels in the plasma were measured with the Oxiselect^TM^ *in vitro* ROS/RNS assay kit (Cell Biolabs, Inc. San Diego, CA), following the manufacturer's instruction and described previously 19, 27. Briefly, 50 µL of the plasma samples or standard were added to a 96-well plate and mixed with 50 µL of catalyst and 100 µL of 2',7'-dichlorodihydrofluorescein diacetate (DCF). After incubation at room temperature for 30 minutes, the fluorescence (Ex480nm/Em530nm) was measured using a Synergy HT Multi-Mode Microplate Reader (Bio-Tek Instruments, Inc., Winooski, VT, USA).

### Western immunoblotting

Protein abundance in heart was measured as previously described 28. Briefly, the middle slices of LV tissues were isolated and homogenized in a lysis buffer followed by centrifugation at 4℃ for 20 minutes at 10000g, and the supernatants were collected. Samples with equal proteins were loaded onto 7.5% polyacrylamide gel with 0.1% sodium dodecyl sulfate and separated by electrophoresis at 100 V for two hours. Proteins were then transferred onto nitrocellulose membranes and incubated with primary antibodies against LC3 (Cell Signaling Technology), Beclin-1 (Cell Signaling Technology), p62 (Cell Signaling Technology), Atg5 (Cell Signaling Technology), GSK3β (Cell Signaling Technology), p-GSK3β (Cell Signaling Technology), respectively. After washing, membranes were incubated with secondary horseradish peroxidase-conjugated antibodies. Proteins were visualized with enhanced chemiluminescence reagents, and blots were exposed to Hyperfilm. Results were quantified with the Kodak electrophoresis documentation and analysis system and Kodak ID image analysis software (Kodak, Rochester, NY). The target protein abundance was normalized to the abundance of GAPDH as a protein loading control.

### Statistical analysis

All data are expressed as the mean ± SEM obtained from the number (n) of experimental animals given. Differences between the groups were compared by Student's t-test or analysis of variance (ANOVA) using the Graph-Pad Prism software (GraphPad Software Version 4, San Diego, CA, USA), where appropriate. For all comparisons, P-values less than 0.05 indicated statistical significance.

## Results

### Effects of chronic nicotine exposure on I/R-induced myocardial infarction and heart function

**Figure 1:**
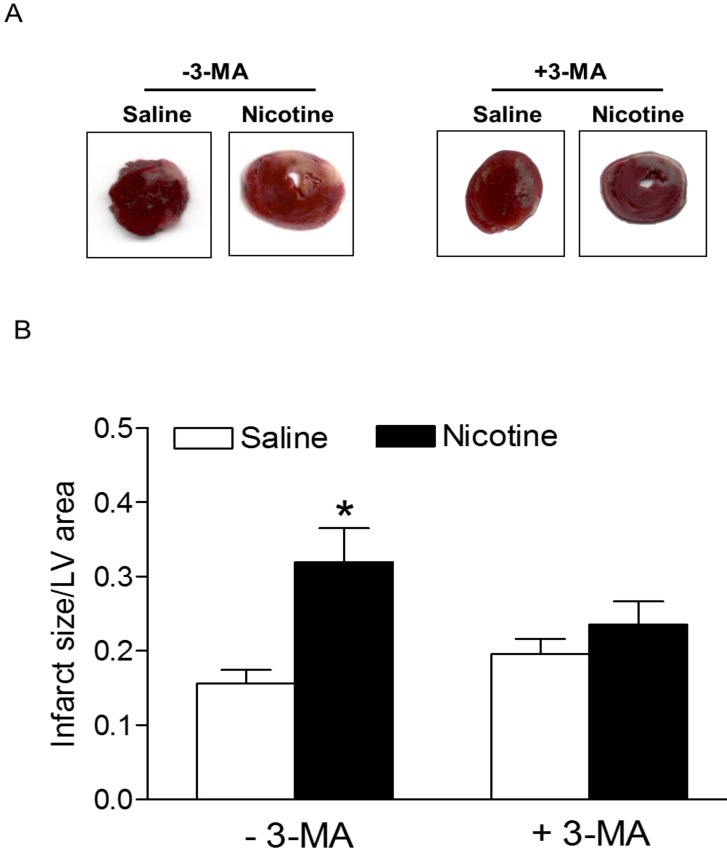
** Nicotine exacerbates on ischemia/reperfusion (I/R)-induced myocardial infarct size and 3-MA reduces it.** Rats from each group were subjected to 45 min of heart ischemia followed by 24 hours of reperfusion. After I/R, the hearts were isolated and their infarct sizes in each rat group were determined with 2% TTC staining (**A**). The bar graph (**B**) showing left ventricle infarct size (infarct area/total LV area) in each rat group. Data are means ± SEM of animals from each group (n=4-5) were analyzed by 2-way ANOVA. *P < 0.05 versus saline control.

**Figure 2:**
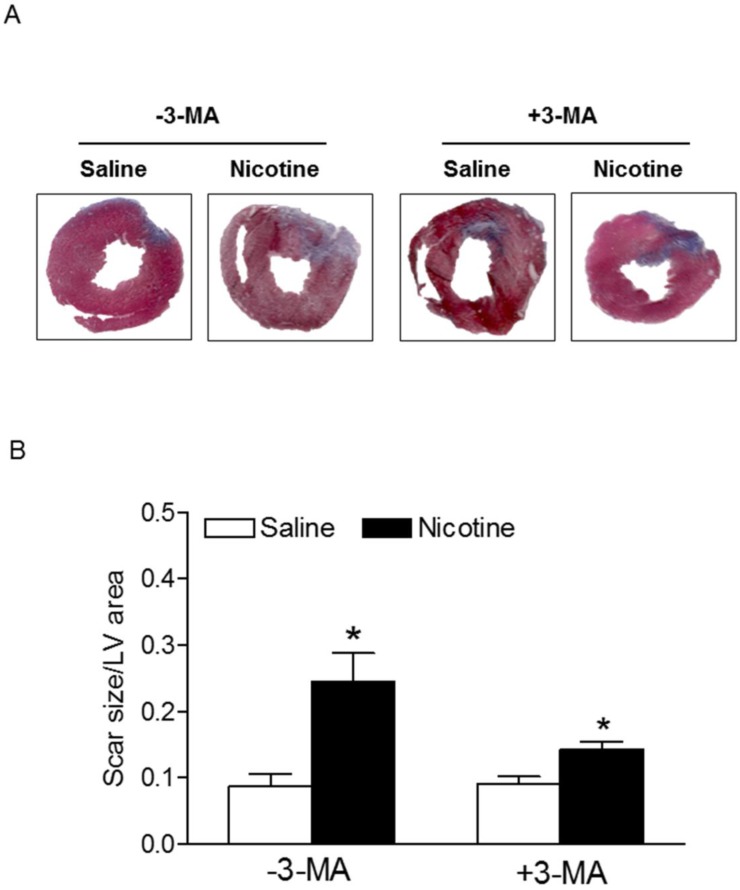
** Nicotine enhances ischemia/reperfusion (I/R)-induced relative myocardial scar area.** Rats from each group were subjected to 45 min of heart ischemia followed by seven days of reperfusion. After I/R, the hearts were isolated and their relative scar areas in each rat group were determined with Masson's trichrome staining (**A**) as described in the Methods section. The bar graph (**B**) showing left ventricle scar size (ratio of scar area to total LV area) in each rat group. Data are means ± SEM of animals from each group (n=4-5) were analyzed by 2-way ANOVA. *P < 0.05 versus saline control.

Chronic nicotine treatment for 30 days had no effect on the body weights of rats (data not shown). Similarly, there was no significant difference in the ratio of heart to body weight between the saline control and nicotine-treated groups (data not shown). As shown in Fig. 1, after 45 minutes of ischemia followed by 24 hours of reperfusion, there was a remarkable increase in myocardial infarction in rats. In the absence of autophagy inhibitor Methyladenine (3-MA), chronic nicotine exposure induced a significant increase in myocardial infarct size as compared with the saline control group under I/R procedure (Fig. 1). However, in the presence of 3-MA (Fig. 1), 3-MA treatment eliminated the difference of infarct size between the saline control and nicotine-treated group under I/R procedure. As shown in Fig. 2, the cardiac scar size after seven days of reperfusion was significantly higher in the nicotine-treated group than the saline control group in the absence of 3-MA (P < 0.05). However, treatment with 3-MA had the trend to attenuate nicotine-mediated cardiac scar size but did not reached significance under I/R procedure (Fig. 2).

**Figure 3:**
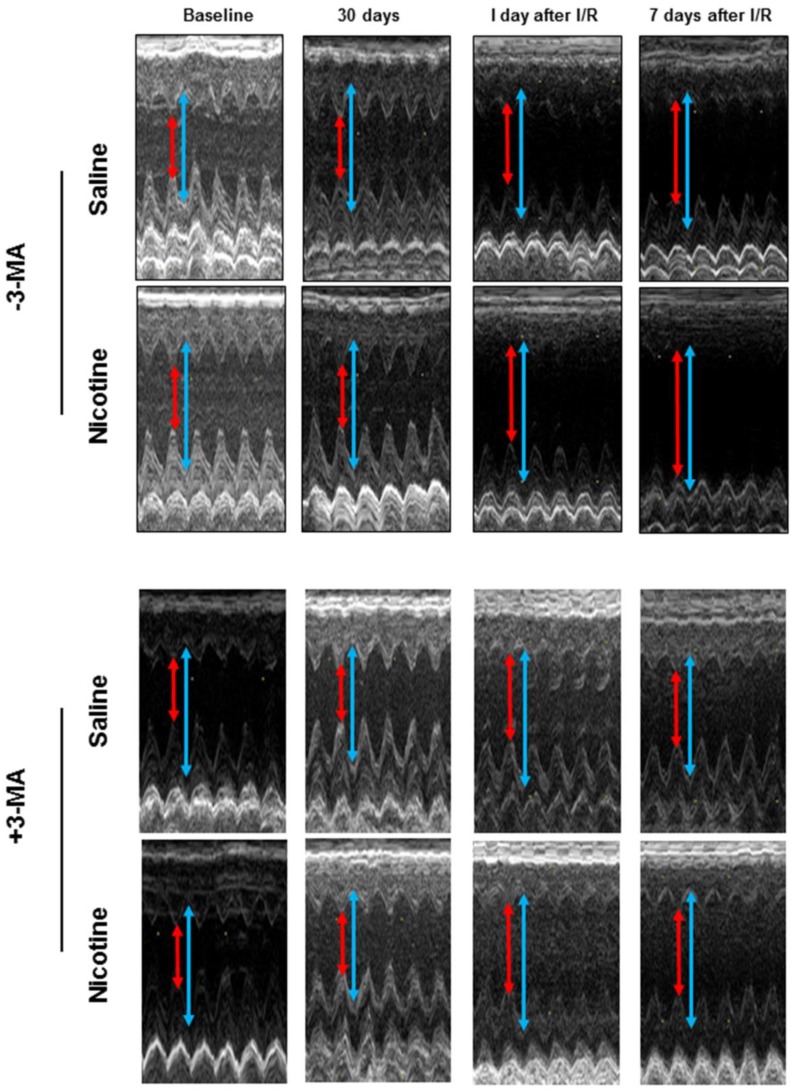
** Echocardiographic evaluation of cardiac function.** Rats were administered with either saline or nicotine for 30 days. Seven days before I/R surgery, 3-MA or saline was administered to rats as described in Methods section. The representative echocardiographic images were obtained from different time periods including at baseline, 30 days of nicotine treatment, one day after I/R, and seven days after I/R. The red arrow represents LV end-systolic dimension (LVESD) and the blue arrow represents LV end-diastolic dimension (LVEDD).

**Figure 4:**
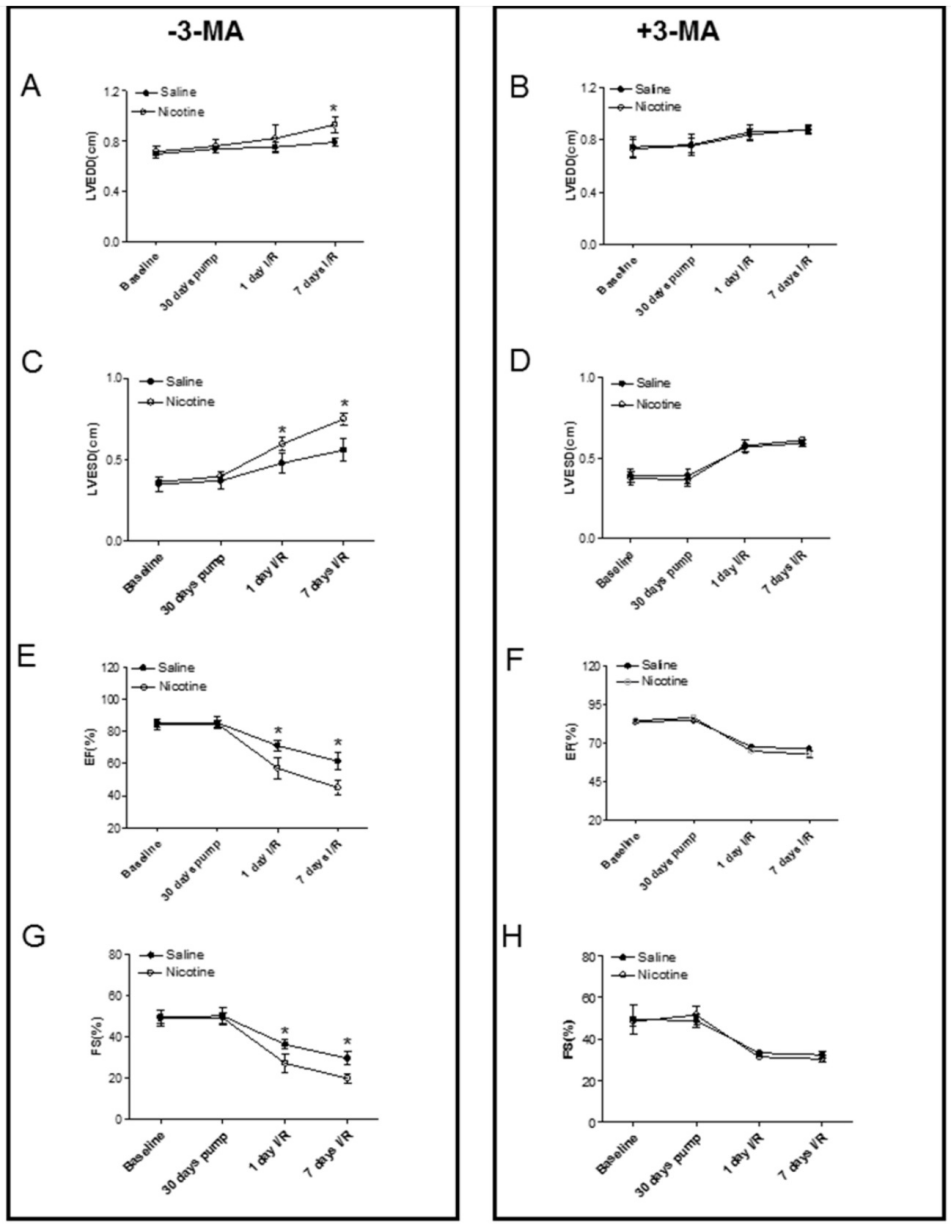
** Effects of nicotine exposure on cardiac function before and after I/R.** The echocardiographic data of both the saline control and nicotine-treated groups were examined at following time periods: before nicotine treatment (baseline), 30 days of nicotine treatment, one day after I/R, and seven days after I/R. The summary data include: left ventricular end-diastolic dimension (LVEDD) in the absence (**A**) and in the presence of (**B**) 3-MA treatment; left ventricular end-systolic dimension (LVESD) in the absence (**C**) and in the presence of (**D**) 3-MA treatment; percent of ejection fraction (EF%) in the absence (**E**) and in the presence of (**F**) 3-MA treatment; percent of fractional shortening (FS%) in the absence (**G**) and in the presence of (**H**) 3-MA treatment. Data are means ± SEM of animals from each group (n=4-5) were analyzed by 2-way ANOVA. *P < 0.05 versus saline control.

Fig. 3 shows typical echocardiographic images at different time periods in both saline control and nicotine-treated groups in the absence and presence of 3-MA treatment. Fig. 4 shows the summary data obtained from the echocardiography analysis. As shown in Fig 4, there were no significant differences of left ventricular end-diastolic dimension (LVEDD), LV end-systolic dimension (LVESD), ejection fraction (EF) and fractional shortening (FS) at baseline and at 30 days of nicotine treatment between the saline control and nicotine-treated groups. However, after ischemia/reperfusion (I/R), the values of LVEDD (Fig. 4A) and LVESD (Fig. 4C) were reperfusion time-dependently increased. In addition, the values of EF (Fig. 4E) and FS (Fig. 4G) were reperfusion time-dependently decreased in nicotine-treated animals as compared to the saline control animals under I/R procedure. More importantly, treatment with autophagy inhibitor, 3-MA, abolished the nicotine-mediated changes of LVEDD (Fig. 4B), LVESD (Fig. 4D), EF (Fig. 4F), and FS (Fig. 4H) in the animals under I/R procedure.

### Effects of chronic nicotine exposure on autophagy-associated biomarkers

**Figure 5:**
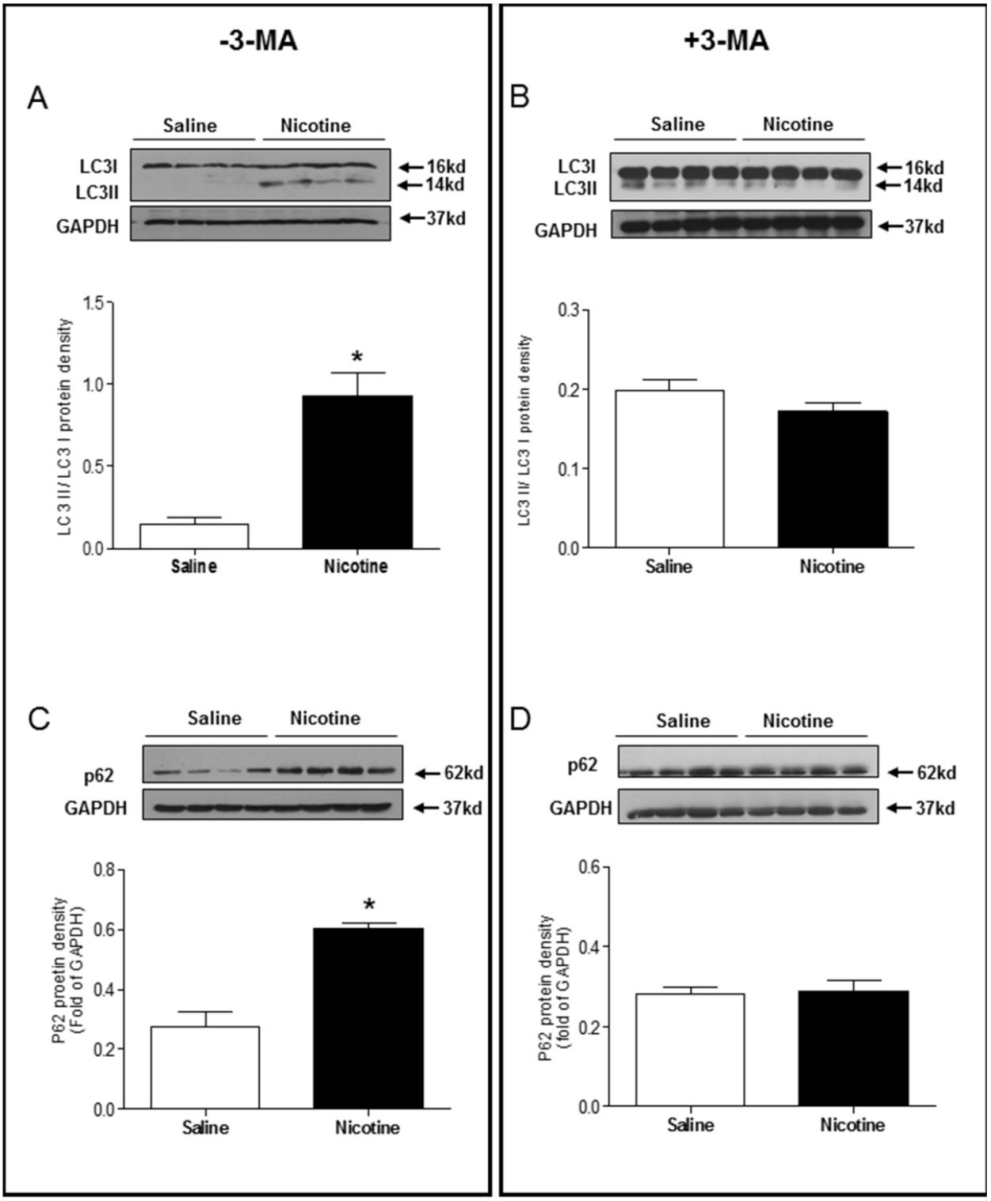
** Effects of nicotine exposure on the protein expressions of LC3 II/LC3 I and p62.** Rats from each group were subjected to 45 minutes of heart ischemia and seven days of reperfusion. After I/R, the hearts were isolated and the protein abundance in the left ventricle tissue was determined by Western blot analysis. LC3 II/LC3 I protein density in the absence of 3-MA (**A**) and in the presence of 3-MA (**B**) treatment. p62 protein density in the absence of 3-MA (**C**) and in the presence of 3-MA (**D**) treatment. Data are means ± SEM (n=4 animals/groups). *P < 0.05 vs. control, as determined by Student's t-test.

**Figure 6:**
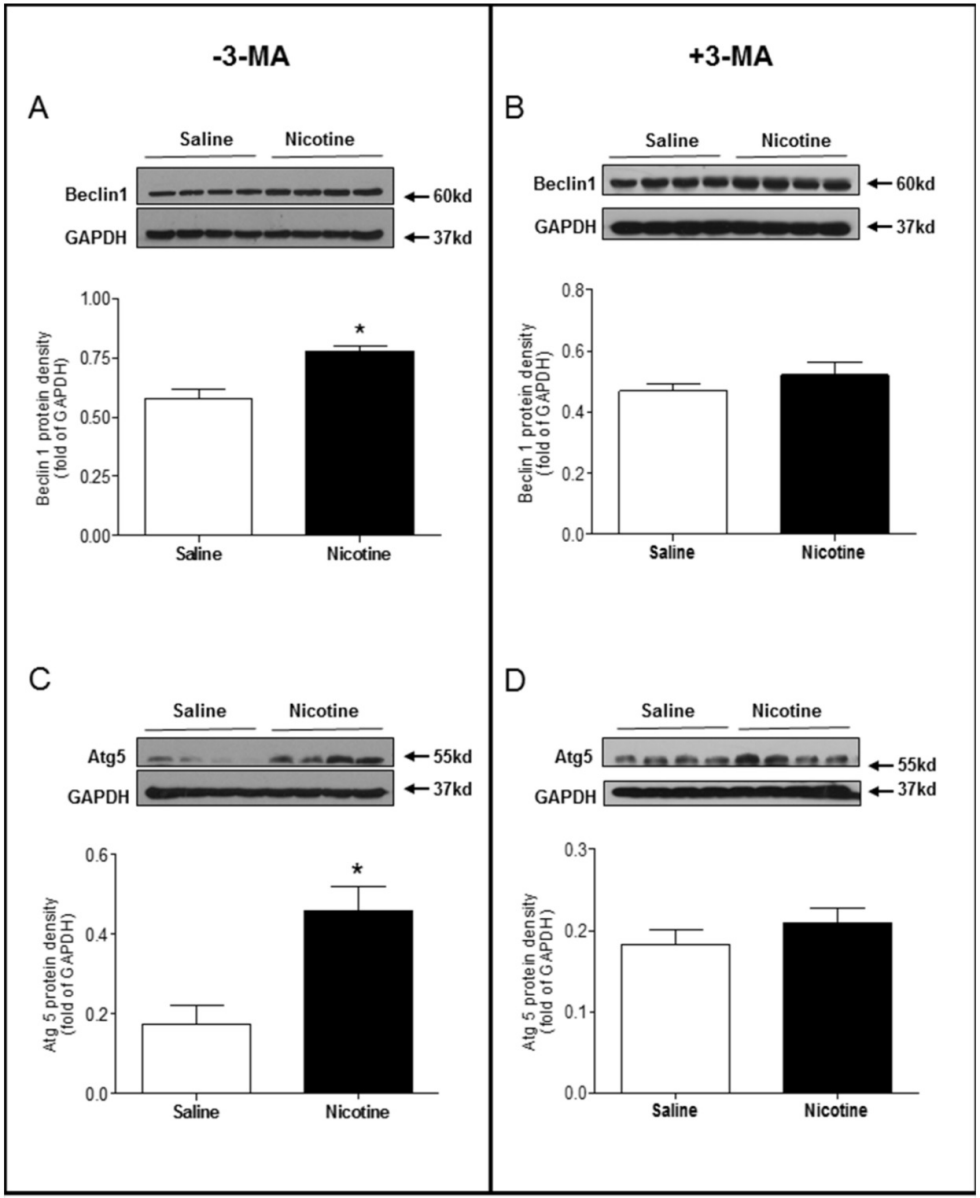
** Effects of nicotine exposure on the protein expressions of Beclin1 and Atg5.** Rats from each group were subjected to 45 minutes of heart ischemia and seven days of reperfusion. After I/R, the hearts were isolated and the protein abundance in the left ventricle tissue was determined by Western blot analysis. Beclin 1 protein density in the absence of 3-MA (**A**) and in the presence of 3-MA (**B**) treatment. Atg5 protein density in the absence of 3-MA (**C**) and in the presence of 3-MA (**D**) treatment. Data are means ± SEM (n=4 animals/groups). *P < 0.05 vs. control, as determined by Student's t-test.

Chronic nicotine exposure significantly enhanced the LC3 II/LC3 I protein expression ratio (Fig. 5A) and p62 protein expression levels (Fig. 5C) in the LV tissues as compared with saline control group in the absence of 3-MA under I/R procedure. However, in the presence of 3-MA, the ratio of LC3 II/LC3 I protein (Fig 5B) and the levels of p62 protein (Fig. 5D) in the LV tissues were not significantly different between the saline control and nicotine-treated rats under I/R procedure. Similarly, the protein levels of Beclin 1 (Fig. 6A) and Atg5 (Fig. 6C) in the LV tissues were higher in the nicotine-treated rats than those in the saline control group, and 3-MA treatment also eliminated these differences between the saline control and nicotine exposed groups under I/R procedure (Fig. 6B and 6D).

### Effects of chronic nicotine exposure on reactive oxygen species (ROS) production

**Figure 7:**
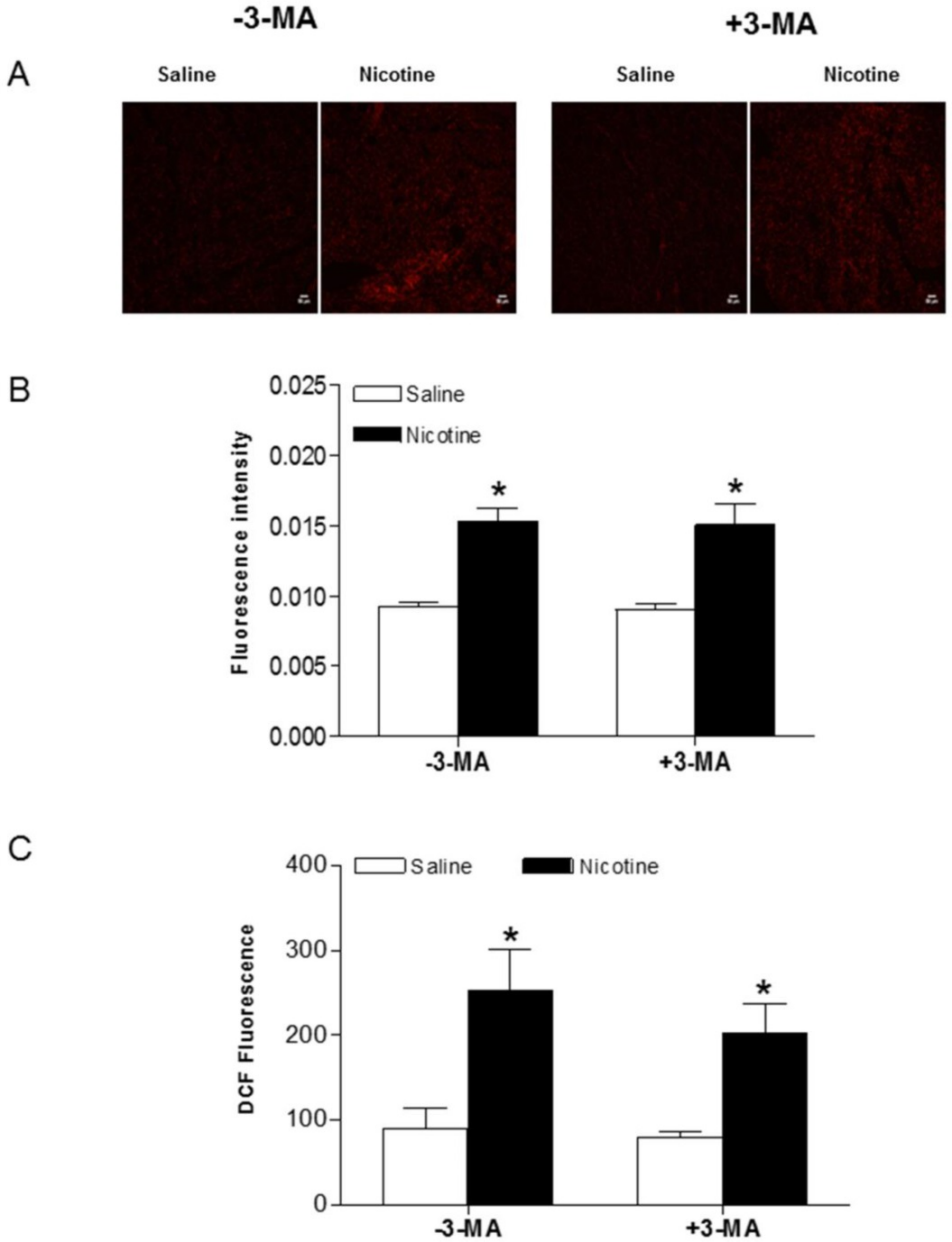
** Effects of nicotine exposure on cardiac and plasma ROS production.** Rats from each group were subjected to 45 minutes of heart ischemia and seven days of reperfusion. After I/R, plasma from each rat was collected and LV segments were cut into 10-µm thick sections as described in Methods section. HE fluorescence from each LV section was used to image (**A**) and analyze ROS production (**B**) *in situ* in the absence and presence of 3-MA. Plasma ROS levels were measured using *in vitro* ROS/RNS assay kit (**C**). Data are means ± SEM of tissue from 4 animals from each group. Scale bar is 50 µm. *P < 0.05 versus saline control, as determined by Student's t-test.

To determine whether chronic nicotine exposure causes oxidative damage in the heart, production of superoxide anion (O_2_^-^) was assessed by the oxidative fluorescent dye HE. As shown in Fig. 7A and B, chronic nicotine exposure increased the production of O_2_^-^ in the LV tissues as compared with the saline control groups in the absence of 3-MA under I/R procedure. Interestingly, 3-MA treatment did not affect nicotine-mediated increase in O_2_^-^ production under I/R procedure. In addition, we determined the ROS production in the plasma using a ROS assay kit under I/R procedure. As shown in Fig. 7C, the plasma ROS levels in the nicotine-treated animals were substantially higher than those in the saline control animals, and 3-MA treatment had no effect on nicotine-mediated ROS production in the plasma.

### Effects of chronic nicotine exposure on cardiac glycogen synthase kinase 3 beta (GSK3β) activity

**Figure 8:**
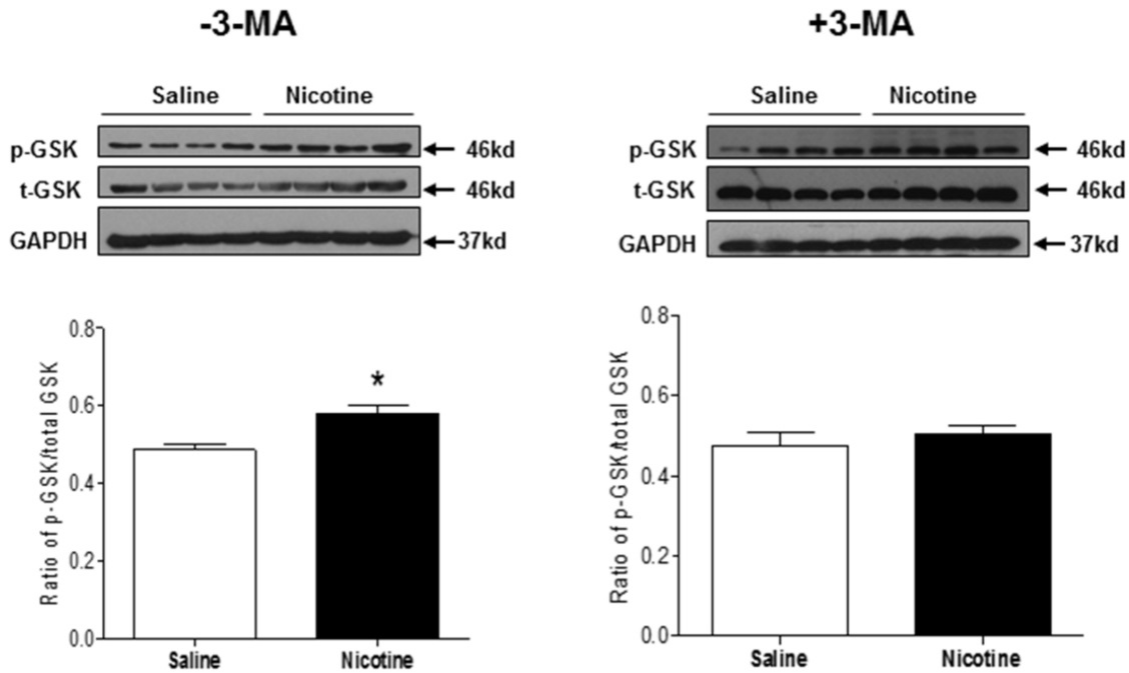
** Effects of nicotine exposure on the protein expressions of total GSK3β and phosphorylation of GSK3β (ser9).** Rats from each group were subjected to 45 minutes of heart ischemia and seven days of reperfusion. After I/R, the hearts were isolated. The protein abundances of p-GSK3β and total GSK3β in the left ventricle tissues were determined in the absence of 3-MA and in the presence of 3-MA. Data are means ± SEM (n=4 animals/groups). *P < 0.05 vs. control, as determined by Student's t-test.

GSK3β is a master regulator of cardiac cell growth and death, which is highly involved in the setting of heart ischemic injury. To investigate the role of GSK3β signaling in mediating cardiac dysfunction in response to nicotine exposure, the phosphorylation levels of GSK3β at serine 9 and total GSK3β protein expression were determined by Western blot analysis. As shown in Fig. 8, chronic nicotine exposure had no significant effect on GSK3β protein expression, but enhanced the ratio of serine 9 phosphorylation of GSK3β to total GSK3β protein expression in the LV tissues as compared with the control groups under I/R procedure. In addition, 3-MA treatment eliminated the difference of the ratio of serine 9 phosphorylation of GSK3β to total GSK3β protein expression between the control and nicotine-treated animals under I/R procedure.

## Discussion

Epidemiological and animal studies have shown that tobacco smoking increases the risk of ischemic heart disease 3, 29, and suggest that nicotine contributes to myocardial ischemia 3. The present study provides further direct evidence that chronic nicotine exposure increases myocardial infarct size and heart dysfunction in a rat model of ischemia/reperfusion. The major findings in the present study are: (1) chronic nicotine exposure increased cardiac I/R injury and resulted in cardiac dysfunction *in vivo*, which was associated with a significant increase in autophagy biomarkers, oxidative stress-related ROS, and ischemia-related protein GSK3β; (2) inhibition of autophagy with an autophagy inhibitor normalized the aberrant autophagy level and reversed the increased vulnerability to heart I/R injury and dysfunction. These findings suggest a potentially novel therapeutic strategy of anti-autophagy for the treatment of smoking/nicotine-mediated heart ischemic disease.

Our data indicated that the ratio of heart weight to body weight was not changed between the control and nicotine-treated groups. In addition, although we did not measure the ratio of heart weight/body weight after seven days of reperfusion, our echocardiographic observations indicated that there were no differences of LVPWd (data not shown) between the nicotine-treated and control groups. These data suggest that nicotine does not induce cardiac hypertrophy at both resting and I/R-challenged condition. The present study showed that chronic nicotine exposure had no significant effect on pre-ischemic baseline values of heart function determined by echocardiography but significantly increased LV myocardial infarct size and impaired LV function after ischemia/reperfusion in rats. The data suggests that chronic nicotine exposure does not affect heart function at normal physiologic conditions but alters the heart function when it encounters an ischemic stress challenge. Previous studies in human and animal models have reported that cigarette smoking is one of the top risk factors of ischemic heart disease 3, 4, 6, 29. Furthermore, there were positive relations between smoking-mediated myocardial infarct size and nicotine concentration in the animal model 3. The present findings that chronic nicotine exposure enhanced I/R-induced myocardial infarct and scar size, suggest that nicotine is a key factor in cigarette smoking-induced cardiac infarction and heart dysfunction. As a ganglionic agonist, nicotine can bind the nicotinic Ach receptor and stimulate neurotransmitter, catecholamine release. Previous studies have demonstrated that cigarette smoking significantly increases plasma norepinephrine and epinephrine levels, which are stimulated by nicotine 5, 6. Increased circulating concentrations of catecholamines enhance the pulse rate, blood pressure, and ischemic heart injury 5, 6, 28. Therefore, it is likely that cigarette smoking/nicotine exposure-associated increments in ischemic heart infarction are mediated through adrenergic mechanisms by releasing catecholamine. Indeed, our previous studies in a similar pregnant rat model have demonstrated that maternal nicotine exposure during pregnancy enhances norepinephrine levels in the fetal heart, resulting in development of heart ischemia-sensitive phenotype 30.

The molecular mechanisms underlying nicotine-mediated increase in ischemic injury and heart dysfunction are still largely unknown. In the present study, we observed that chronic nicotine exposure enhanced autophagy biomarkers (such as LC3II, p62, Beclin 1, and Atg 5) protein abundance in cardiac tissues. Consistent with our observation, other studies have shown that nicotine exposure increases autophagy activation in human fibroblast cells 31, human cancer colon cells 32, and bronchial epithelial cells 13. These findings suggest that nicotine is one of the key activators of autophagy. Growing evidence has shown an important role of autophagy in the pathophysiological process of ischemic heart injury 33. However, whether autophagy serves a beneficial or detrimental role in the myocardium depends on the level of autophagy activation and the context in which it is induced 8, 10, 11. It has been reported that upregulation of autophagy in the setting of mild ischemia provides a protective effect that promotes functional recovery of the heart. However, there is also strong evidence that excessive autophagy contributes to myocyte death and heart dysfunction 11. Our present findings that treatment with 3-MA significantly inhibited nicotine-mediated autophagy and eliminated the difference of I/R-induced cardiac injury and dysfunction between the saline control and nicotine-treated rats, suggest that nicotine-mediated heightened autophagy is one of the key molecular mechanisms contributing to ischemic heart injury and dysfunction. Consistent with our findings, a previous study has shown that enhanced autophagy leads to accumulation of autophagosomes and cardiac contractile dysfunction, and inhibition of autophagy with 3-MA treatment prevents heart injury 34. In addition, other studies in rat primary cardiac myocytes *in vitro* have demonstrated that treatment of 3-MA or knockdown of Beclin 1 inhibits ischemia/ reperfusion-induced autophagy, leading to reduced cardiac injury and enhanced cardiac myocyte survival 35. In contrast to these finding, our current data (Fig. 1 and 2) indicated that *in vivo* treatment of 3-MA had no effect on I/R-induced heart injury in saline control animals. This discrepant effect of 3-MA between *in vitro* cardiac myocytes and *in vivo* heart may suggest that basal autophagy dose not play a key role in I/R-induced heart injury in animals at physiologic condition. However, our present findings that that treatment with 3-MA significantly inhibited I/R-induced heart infarct size in nicotine-treated animals and eliminated the difference of I/R-induced cardiac infarct size and dysfunction between the saline control and nicotine-treated rats, suggest that the excess of autophagy plays an important role in nicotine-mediated I/R-induced heart ischemic injury and dysfunction. Taken together, our current findings with others suggest a causal effect of autophagy in the setting of ischemic heart injury and dysfunction under pathologic condition. In addition, we need keep in mind that autophagy signaling pathway may be not the only signaling pathway involved nicotine-mediated cardiac dysfunction, other signaling pathways such as apoptosis may also be involved.

The process of autophagy is complex and mainly comprised of the following stages: initiation, nucleation, expansion, maturation, and degradation. Numerous autophagy-related (Atg) genes and signaling molecules have been identified and are sequentially activated to regulate different stages of autophagy. There are three classes of PtdIns3K and phosphatidylinositol 3-Kinases (PI3Ks) involved in the regulation of autophagy. Class I PI3K responds to growth factor signaling and produces PtdIns(3,4,5)P3 that negatively regulates autophagy via the AKT-mTOR complex, whereas the class III PI3K, as well as class II PI3K, contributes to the initiation and progression of autophagy. Beclin 1 is one of the major genes involved in the class III PI3K complex signaling 36. The formation of autophagosome is often initiated with Beclin1 as an internal stimulus followed by several autophagy related proteins including Atg5 37. In the present study, our data indicated that nicotine exposure enhanced the protein levels of Beclin1 and Atg5 in the LV tissues, which were reversed by the treatment of 3-MA. Consistent with our findings, previous studies demonstrated that cigarette smoking induces autophagy via increasing Beclin1 and Atg5 gene expressions 38. These data suggest that activation of the class III PI3K signaling pathway is one of the important mechanisms underlying nicotine-induced cardiac autophagy. In addition to the regulation of class III PI3K pathway, our present findings that nicotine exposure significantly increased p62 protein levels in ischemic heart tissues, which was abrogated by 3-MA treatment, suggest that p62 also play a key role in nicotine-mediated autophagy. Similarly, previous studies have demonstrated that nicotine increases co-expression of impaired autophagy marker, SQSTM1/p62 and ubiquitin 13. It is well known that ubiquitinated cargo including injury organelles and potentially toxic protein aggregates is delivered to autophagosomes by the receptor protein p62 39, 40. The interaction between autophagosomes and SQSTM1/p62 has been observed in smokers' alveolar macrophages 41. The formation of autophagosome is often initiated with Beclin1 as an internal stimulus followed by several autophagy related proteins including Atg5 37. Thus, we speculate that Beclin 1/Atg5/p62/LC3II signaling may be one of the major signaling pathways underlying nicotine-mediated autophagy and ischemic heart injury.

It has been shown that cigarette smoking increases the sensitivity of hearts to ischemia/ reperfusion injury associated with increased production of oxygen free radicals 42. Consistent with this study, our present study also showed an increase in ROS production in both plasma and cardiac tissue of the rat exposed to nicotine. Furthermore, our previous studies have demonstrated that inhibition of ROS reverses perinatal nicotine-mediated ischemic heart injury and heart dysfunction 43. These studies suggest that oxidative stress plays a key role in the aberrant development of heart ischemia-sensitive phenotype in response to cigarette smoking/nicotine exposure. The relationship between ROS and autophagy has been demonstrated in several other systems 44, 45, but the cause-and-effect relation is controversial. For example, amino acid deprivation induces the formation of ROS in mitochondria in a Class III PI3K-dependent manner 45. On the contrary, p62 overexpression increases ROS and accelerates tumorigenesis 46. Although molecular mechanisms underlying nicotine-induced excessive autophagy in the heart are not fully understood, previous studies have shown that stress-induced ROS is essential for the induction of autophagy 45. We speculate that nicotine-mediated ROS release may be one of the key mechanism underlying nicotine-induced excessive autophagy. Indeed, our current study found that 3-MA treatment did not affect nicotine-mediated ROS production. Consistent with our finding, previous studies have also demonstrated that 3-MA has no effect on ROS production in starvation-induced or dexamethasone-induced autophagy 47. These findings suggest that ROS is the up-stream signaling of autophagy in our animal model. Future studies are needed to employ a specific ROS scavenger or antioxidant to confirm whether the heightened ROS is one of the key mechanisms underlying nicotine-induced autophagy.

GSK3β gene plays a significant role in embryonic cardiomyocyte development. Recent studies demonstrate a functional role of GSK3β in negative modulation of cardiac hypertrophy and in the setting of cardiac ischemic disease 48-50. GSK3β is a unique protein kinase. The dephosphorylated GSK3β is in active form, but GSK3β will be inactivated when it is phosphorylated at the serine 9 residue by other factors 51, 52. In present study, we demonstrated that chronic nicotine exposure enhanced the phosphorylation levels of GSK3β at the serine 9 residue as compared to the saline controls. This suggests that the enhanced phosphorylation of GSK3β may lead to a decrease in cardiac GSK3β activity. In addition, our present data that treatment of 3-MA eliminated the difference of the phosphorylation level of GSK3β between the saline control and nicotine-treated animals, suggests that the enhanced phosphorylation of GSK3β is at least partially regulated through an autophagy-dependent signaling pathway.

In summary, the present study provides new evidence that excess autophagy is one of the key molecular signaling linkers between nicotine exposure and the development of the heart ischemic-sensitive phenotype. Our data indicated that nicotine exposure stimulated oxidative stress and enhanced cardiac autophagy biomarkers, resulting in increased ischemic heart infarct size and heart dysfunction. Of importance, inhibition of autophagy via 3-MA rescued nicotine-mediated pathological effects and heart dysfunctions. These findings not only provide novel evidence regarding the molecular mechanisms underlying nicotine-induced ischemic heart injury and dysfunction, but also suggest a potential therapeutic molecular target of autophagy involved in the setting of ischemic heart disease.

## Future studies

In our present study we have demonstrated that chronic nicotine exposure enhances I/R-induced heart dysfunction associated with an excessive autophagy flux. However, one of the limitations for this study is unknown whether the induction of the autophagy proteins is directly regulated by nicotine exposure or I/R procedure. Therefore, in our future study we should measure the basal levels of autophagy influx in the control and nicotine-treated animals to see whether nicotine-mediated pre-existed increase of autophagy in the heart would exacerbate the ischemic/reperfusion injury. While infarct size and cardiac function are good indicators of myocardial ischemia/reperfusion injury, other indicators such as Troponin I concentration can further support the claim. Thus, in our future studies, we will investigate whether nicotine exposure affects Troponin I expression in response to I/R. It's well known that 3-MA is a selective autophagy/PI3K inhibitor. However, 3-MA could induce potential non-specific effects on heart function. It depends on the patho-physiologic condition of the animals and dosage of 3-MA. For instance, previous studies have reported that 3-MA has dual role in modulation of autophagy and induces an autophagic promotion effect under nutrient-rich condition 53. Moreover, 3-MA can inhibit tumor metastasis in an autophagy-independent manner 54. Furthermore, 3-MA has an anti-inflammatory effect in atherosclerosis 55. Therefore, further investigation is needed to unmask the diverse regulations of 3-MA on nicotine-mediated heart I/R injury and dysfunction.

## Abbreviations

3-MA: 3-methyladenine
I/R: ischemia/reperfusion
LC3: microtubule-associated protein light chain 3
P62: sequestosome 1
Atg5: autophagy related 5
ROS: reactive oxygen species
RNS: reactive nitrogen species
GSK3β: glycogen synthase kinase 3 beta
LV: left ventricle
LVEDD: left ventricular end-diastolic dimension
LVESD: left ventricular end-systolic dimension
LVEDV: left ventricular end-diastolic volume
LVESV: left ventricular end-systolic volume
LVPWd: left ventricular posterior wall thickness at end-diastole
EF: ejection fraction
FS: fractional shortening
LAD: left anterior descending artery
TTC: 2,3,5-triphenyltetrazolium chloride
OCT: optimal cutting temperature
HE: hydroethidium
DCF: 2',7'-dichlorodihydrofluorescein diacetate
GAPDH: glyceraldehyde 3-phosphate dehydrogenase
ANOVA: analysis of variance
PI3K: phosphatidylinositol 3-kinases
AKT: protein kinas B
mTOR: mammalian target of rapamycin
